# Reassessing green exercise research: unveiling methodological gaps and pathways for progress

**DOI:** 10.3389/fspor.2024.1449059

**Published:** 2024-11-19

**Authors:** Luca Laezza, Stefano De Dominicis, Margherita Brondino

**Affiliations:** ^1^ Department of Human Sciences, University of Verona, Verona, Italy; ^2^ Department of Nutrition, Exercise and Sports, University of Copenhagen, Copenhagen, Denmark

**Keywords:** green exercise, environmental measures, literature review, nature exposure, physical activity

## Abstract

The present review critically assesses the nexus between physical activity, nature exposure and health benefits by analysing environmental measures in green exercise research. A Cochrane-inspired review of systematic studies exposes methodological gaps, emphasising the scarcity of long-term research and the lack of rigorous designed studies. It calls for more robust, varied research designs and improved environmental metrics. The findings advocate for longitudinal research to better comprehend the mental and physical health benefits of exposure to nature. Embracing an ecological-dynamic perspective is recommended to advance our understanding of the intricate connections between activity, environment, and well-being.

## 1 Introduction

Currently, 55% of the global population resides in urban areas, which is projected to increase to 68% by 2050. The growing trend of urbanisation poses significant public health challenges, including environmental degradation, insufficient infrastructure, scarce green spaces, pervasive air pollution, and a dearth of areas designated for physical activity (PA) (1). Furthermore, the expansion and densification of cities are expected to reduce both the availability and quality of green spaces per capita, which could negatively impact the restorative potential of these areas (2).

However, the health benefits of green spaces in urban environments, such as reduced risk of noncommunicable diseases, have been well-documented (1), and the World Health Organization's “Global Action Plan for Physical Activity 2018–2030” highlights the crucial role of accessible green spaces in fostering environments that support PA (3). Green spaces also offer co-benefits in the context of climate change adaptation, contributing to heat stress reduction and improved water retention while simultaneously promoting mental well-being (2).

Engaging in PA exerts a plethora of physical and psychological benefits, such as reduced risk of chronic cardiovascular and musculoskeletal diseases, hypertension, diabetes, osteoporosis, fractures, and certain types of cancer (4), as well as symptoms of anxiety and depression (5–8). However, despite recognising sedentary lifestyles as a global public health issue, efforts to implement effective programs and policies promoting PA across populations have been inadequate (9).

Within such a scenario, exposure to green spaces is linked to lower depression levels (10), higher perceived mental health, lower mortality (11), and improved affective states (12), reduced stress and more rapid recovery from it (13, 14).

### 1.1 Green exercise

Researchers (15) have advocated for the added physical and mental health benefits of green exercise (GE) — i.e., exercising while being immersed in nature (16). GE condenses the psychological, physiological, and social gains obtained from PA while acknowledging the positive impact of exposure to nature (17). The main argument is that the benefits of PA and exposure to nature are amplified when combined, offering synergistic health effects (16). Accordingly, the positive impact of nature on health and well-being can be categorised into three domains: mitigating damage (mitigation), fostering the recovery of resources (restoration), and enhancing capabilities (instoration) (18).

### 1.2 Restoration domain

Beyond the mitigation effect of nature on physical and mental health (e.g., better air quality and heat and noise reduction (18–21), its restorative effect has been well documented. In fact, daily demands often deplete personal resources — physiological, psychological, or social — but can be replenished in restorative environments (22, 23). Stress Reduction Theory (SRT) suggests that natural environments, unlike urban ones, elicit immediate stress relief due to an innate positive affective response, leading to quicker recovery (24, 25). Such responses are believed to be evolutionary and are triggered by specific environmental features (e.g., vegetation, water) that signal safety and foster well-being (20, 26).

Attention Restoration Theory (ART) (24, 27, 28) proposes that natural environments aid cognitive function by replenishing attention. It differentiates between involuntary, effortless attention and voluntary, directed attention, with the former automatically activated by nature (27, 29). Four features foster attention restoration, as stated by ART: fascination, being away, extent, and compatibility (30–32).

The Perceptual Fluency Account theory advises that an easier and more effortless visual perception of natural scenes leads to positive evaluations, aiding cognitive restoration and stress reduction (33–35).

Finally, the Relational Restoration Theory posits that replenishing depleted resources involves both individual and relational aspects, including the dynamic interaction between an individual and the environment (36) and interactions within dyads or groups (37).

### 1.3 Instoration domain

**Figure 1:**
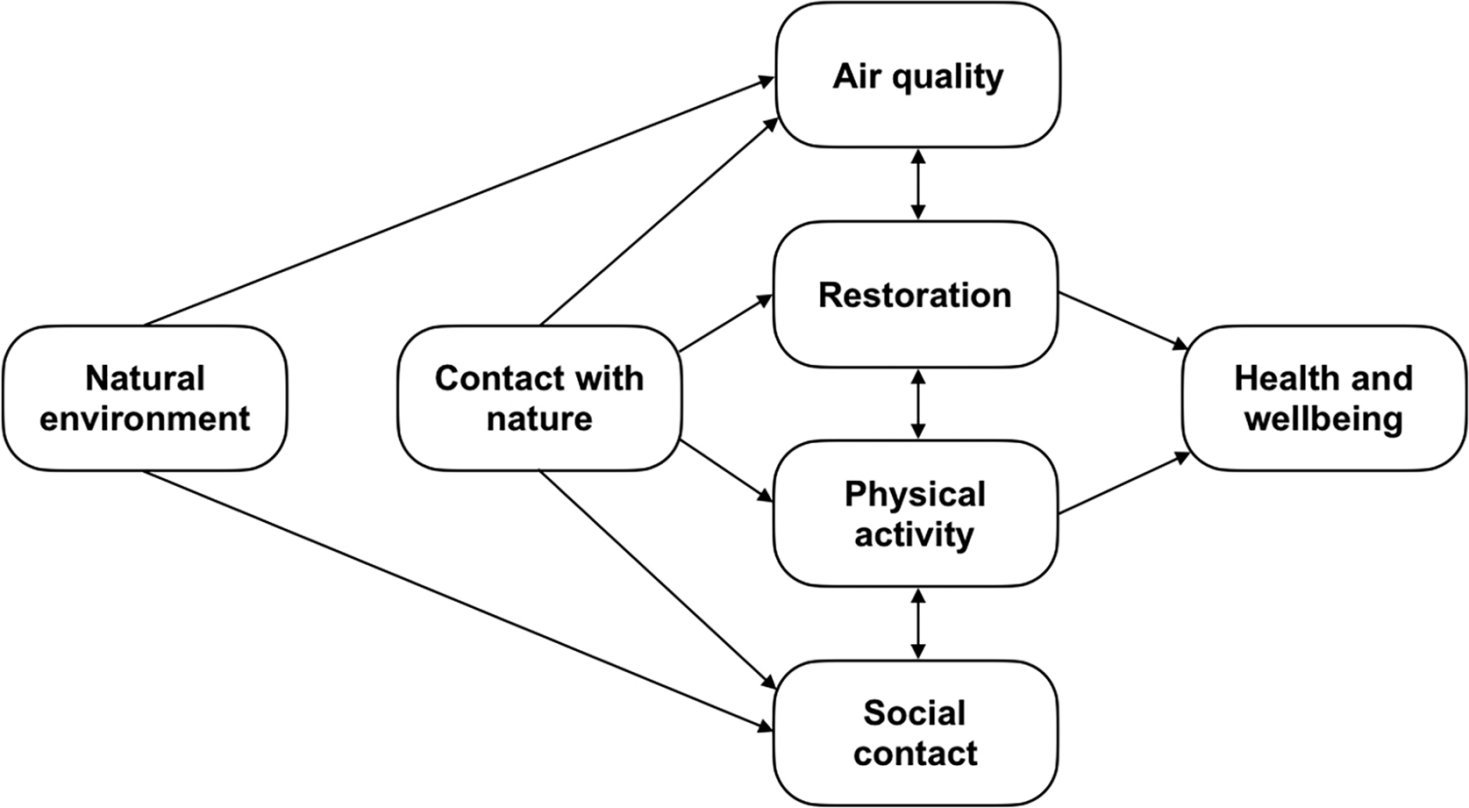
Pathways model of the benefits of natural environments on health and well-being adapted from Hartig et al. (20).

Along with other health-promoting factors like air quality, social interaction, and stress reduction, it is proposed that physical activity (PA) is a means through which natural environments can enhance well-being [Figure 1 (20)]. Green spaces have been shown to encourage an active lifestyle (38), and GE may be particularly restorative, as it allows people to engage with the environment to facilitate resource regeneration (39). However, while a meta-analysis found that green spaces are positively associated with higher physical activity levels in the elderly (40), the overall evidence is mixed and may vary across different population subgroups (41, 42).

Recently, the ecological dynamic perspective has been applied to GE to examine the interdependent relationship between individuals, their environments, and PA. This approach, grounded in Gibson's (43) theory of perception, emphasises behaviour emergence, constraint interaction, and environmental affordances as crucial concepts in understanding health-related behaviours (44–46). Within this framework, Rogerson et al. (17) proposed a two-pathway model, suggesting that exercise not only provides access to green spaces but that these settings can also have direct salutogenic effects, thereby influencing PA and health outcomes.

### 1.4 Methodological issues in green exercise research

GE research encounters theoretical and practical challenges. Evidence supports GE's benefits, yet the relationship between exposure to natural environments and the resulting PA remains indistinct (10). At least three different GE research approaches can be distinguished (47): (1) investigation of the effects of exercise in built environments compared to natural outdoor environments; (2) comparison of outcomes of GE with those of indoor exercise; (3) study of the effects of different visual environments (e.g., showing participants natural or urban videos or images) during indoor exercise. Each approach has limitations and strengths, and comparing their results is challenging (47). Methodological constraints relate to both components of GE: the PA and the environment where it occurs.

#### 1.4.1 Measuring PA

Quantifying the PA necessary for a healthy lifestyle is complex due to behavioural variability (15). In the general population, measuring PA levels largely relies on self-report questionnaires despite their lack of reliability compared to objective measures (48). Researchers commonly use the long form of the validated International Physical Activity Questionnaire (IPAQ-LF) to measure PA levels (49). Nevertheless, research indicates that self-reports can overestimate vigorous PA (50). Discrepancies have also been observed when comparing the IPAQ's short form with accelerometer data, suggesting an overestimation of moderate-intense PA and an underestimation of sedentariness (51, 52).

#### 1.4.2 Measuring nature exposure

A significant methodological gap in studying nature's health effects lies in measuring nature exposure (20). At the epidemiological level, exposure is typically assessed through exposure frequency and duration, the distance of green space from the residence or its amount in the surroundings (53). The Normal Difference Vegetation Index (NDVI) is one of the most widely used metrics for providing data on vegetation density and distribution (54). However, it falls short of differentiating between vegetation types and does not convey the quality of green spaces (18). Studies also utilise Google Street View to estimate the green view index from street-level imagery. This approach falls short in evaluating environmental quality and the interaction of natural features within the activity setting (55, 56). Thus, given the abovementioned fragmented literature, it is paramount to summarise and understand the state-of-the-art to clarify and suggest possible future research directions within the study of GE-environment transactions.

## 2 Review

### 2.1 Scope

Accordingly, this review aims to clarify methodological issues concerning the study of the role of the physical environment in GE research. This analysis uses a framework based on the Cochrane methodology to overview systematic reviews (57). This approach is well-suited for reviewing multiple systematic reviews on a shared topic while addressing questions not initially included in the reviews. Specifically, this overview will: (a) compare study conditions and research approaches, following Barton et al.'s (47) methodological classification; (b) highlight key findings by considering comparison conditions and research designs (e.g., short-term and long-term effects); (c) synthesise the features of green spaces where activities occur, detailing the type of greenspace, objective, and subjective measures of nature exposure.

### 2.2 Method

An electronic literature search was conducted to identify reviews and meta-analyses about PA and exercise interventions in the natural environment. PICOS criteria were drafted to guide the search (see Table 1). A search strategy was developed using keywords and search terms retrieved from papers on the topic. PubMed and Scopus Electronic databases were searched with keywords and search strings organised in blocks (see Table 2). The search was limited to systematic reviews and meta-analyses in English published in the last 15 years. Thus, a narrative synthesis of the included reviews is outlined, as the statistical findings were outside this review's scope (53).

**Table 1:** PICOTS criteria.

Population	Healthy adults
Intervention	PA carried out outdoors or in a natural environment
Comparator	PA carried out indoors or in an urban environment or control activities
Outcome	Physical well-being and performance Psychological health Quality of life
Timing	15 years limit
Design	Experimental studies

**Table 2:** Keywords and research strategy.

PA	(exercis* OR “PA” OR “physical activities” OR “physical exercis*” OR “exercise training” OR “green exercise”)
Environment	(nature OR “natural environment*” OR outdoor OR “green space” OR “green spaces” OR greenspace)
Outcome	(health OR “mental health” OR “physical health” OR “mental well-being” OR “physical well-being”)

## 3 Results

The search revealed 1,170 references. Two more articles were added by hand, searching as relevant to the topic. After duplicates were removed, 1,008 references were found as potentially eligible. After screening the title and abstracts, 958 references were excluded for reasons. The remaining 49 articles were full-text screened, and six reviews were retained.

### 3.1 Comparison conditions

The included studies employed various methods to compare GE interventions (Table 3). Bowler et al. (58) included studies comparing GE to indoor exercise or urban outdoor exercise. Indoor environments were gyms and laboratories, whereas outdoor non-green environments involved streets and residential areas. Only a few studies compared the same activity in each environment. Thompson-Coon et al. (59) conducted a review to compare the effects of GE vs. indoor exercise. Indoor exercise was conducted in gyms, fitness facilities, laboratories, and a shopping centre. Two studies involved virtual reality as the green setting. Lahart et al. (60) conducted a systematic review to investigate the effects of exercise performed in a natural outdoor or virtual environment compared with indoor exercise. Four trials lacked a non-green condition, wherein participants engaged in indoor exercise while watching a video of the same route being performed outdoors. Yen et al. (61) conducted a systematic review and meta-analysis to examine the long-term influence of PA in natural settings on quality of life. Two studies involved PA intervention in aquatic environments. Only three trials compared green or blue exercise with PA conducted in non-green settings; the remaining studies involved other types of control groups such as occupational therapy, social activities, or waitlists. Wicks et al. (62) conducted a systematic review to compare the psychological health effects of GE with exercise in urban environments. Marini et al. (62) conducted a systematic review to explore the effects of PA interventions in green and blue environments. However, four out of six studies lacked a comparison condition.

**Table 3:** Summary of the comparison condition, type of activity, and green exercise environments of the interventions included in the considered reviews.

Review	Studies	Green conditions	Comparation conditions	Activities	Green condition environments
Bowler et al. (58)	25	Natural outdoor	Outdoor urban environments Indoor	Walking Running Wilderness backpacking Gardening Passive-sedentary activity mixed activities	Parks University campuses Nature reserve Wildlife reserve Wilderness Forest garden
Thompson Coon et al. (59)	11	Natural outdoor	Indoor	Running Walking	University/college campus sidewalks/walking Paths Forest Country park Described as “outdoor”, no further details are provided
Lahart et al. (60)	28	Natural outdoor indoor virtual natural	Indoor	Walking Running Cycling Dancing Combined strength and aerobic training	Woodland Gardens parks
Yen et al. (61)	8	Natural outdoor	Urban outdoor Indoor Control group with no activity	Aerobic training Cycling Walking Hiking Free activities	Parks urban green areas Sea Beach Marina
Wicks et al. (62)	24	Natural outdoor	Urban outdoor	Running Walking	Forest Woodland Grasslands Regenerated landfill Nature reserve Urban park Bamboo forest
Marini et al. (63)	6		Urban outdoor No activity or daily routine	Walking strength and resistance training	Urban park Outdoor fitness station Exercise parks Blue and urban environments

### 3.2 Green exercise effects

Most of the reviews focused on the short-term effects of GE, reflecting a lack of long-term studies (for detailed findings, see Table 4).

**Table 4:** Summary of the characteristics of the reviews and their main findings.

Review (year)	Studies	Short-term findings	Long-term findings
Bowler et al. (2010) (58)	25	Reduced negative emotions (anger, fatigue, sadness). Less consistent results on anxiety and tranquillity. A positive small effect was also found on tests of attention. Confidence intervals overlapped zero after adjusting for pre-test measurements on attention and fatigue. Feelings of tranquillity after exposure to nature were more positive than after exposure to an outdoor built environment, but not in comparison to an indoor environment. Comparing the outcomes before and after the activity, positive changes were found in feelings of anxiety, energy, anger, fatigue, and sadness.	NA
Thompson Coon et al. (2011) (59)	11	Walking in a natural environment compared to walking indoors: greater feelings of revitalisation and positive engagement. Decreased tension, confusion, anger, and depression and increased energy. However, the results suggested that feelings of calmness and tranquillity may be decreased following outdoor exercise. Participants reported greater enjoyment and satisfaction with the outdoor activity and intended to repeat the activity later. After running outdoors, compared to running indoors, less anxiety, depression, anger, hostility, and fatigue were reported. However, no benefits were reported in the other two studies involving running as PA.	NA
Lahart et al. (2019) (60)	28	Compared with indoor exercise, acute bouts of outdoor green exercise may favourably influence affective valence and enjoyment but not emotion, perceived exertion, exercise intensity, and biological markers.	In a meta-analysis of three longitudinal trials, the only statistical finding was slightly lower post-intervention perceived exertion with green vs. indoor exercise.
Yen et al. (2021) (61)	8	Na	Green and blue exercise had no significant effect on the general QoL. Green and blue PA revealed a small to moderate significant impact on physical and a small and significant impact on mental health
Wicks et al. (2022) (62)	24	Meta-analysis showed large or moderate effect sizes were obtained for anxiety, fatigue, and positive affect, but considerable heterogeneity was also evident. For vigour, a large effect favouring the natural environment had low heterogeneity. A moderate effect was found for anger, with low heterogeneity. The meta-analysis for depression revealed a small effect in favour of the natural environment but with considerable heterogeneity.	NA
Marini et al. (2022) (63)	6	PA interventions carried out in an outdoor green–blue space natural environment can have a positive impact on a healthy population, both after a few weeks of intervention or after several weeks and can be an effective strategy to enhance and promote healthy lifestyles	NA

#### 3.2.1 Short-term effects

Bowler et al. (58) found that GE yielded greater benefits for self-reported emotions, particularly in reducing negative emotions such as sadness and anger, compared to exercise in non-natural outdoor settings. While the studies in their review examined GE's effects on attention, cardiovascular health, immune system functioning, and PA levels, the benefits were modest. Tranquillity levels were significantly higher after exposure to natural settings than after urban outdoor environments, but not compared to indoor exercise. Thompson-Coon et al. (59) compared the effects of PA in natural environments vs. indoors on psycho-physical well-being, primarily focusing on running and walking activities. Walking in natural settings showed significant improvements in various mood aspects, such as enjoyment, satisfaction, and intentions to repeat the outdoor activity. Running outdoors was associated with lower anxiety, depression, anger, hostility, and fatigue compared to indoor running, although two studies did not report benefits. Due to study heterogeneity and small sample sizes, the authors refrained from drawing conclusive evidence in support of GE. A meta-analysis by Lahart et al. (60) showed mixed results of GE benefits compared to exercising in artificial or indoor environments. Significant short-term effects were found on the affective value and enjoyment of PA. In Wicks and colleagues’ review (62), twenty-two out of twenty-four primary studies examined the short-term effects of GE. In most of the studies (*n* s= 22), participants engaged in walking. Six meta-analyses with data from nine primary studies showed a significant effect of GE for the six outcomes investigated, though the heterogeneity of the results was considerable (Table 5).

**Table 5:** Results of meta-analyses conducted by wicks and colleagues (2022).

Outcome	Participants (studies)	Effect estimate	I^2^
Anxiety	720 (7)	−6.59	91%
Fatigue	697 (5)	−1.98	79%
Positive affect	115 (2)	0.59	92%
Vigor	697 (5)	3.28	15%
Anger/hostility	697 (5)	−0.57	30%
Depression	697 (5)	−0.34	74%

#### 3.2.2 Long-term effects

Upon excluding studies featured in multiple reviews, it was found that 26% of the primary studies delved into the long-term effects of GE. Interventions spanned from 10 weeks to one year. Within this subset, two observational studies focused on clinical populations (58). In three long-term trials, GE was compared with indoor exercise with a similar training volume (60). The meta-analysis investigated the long-term impacts of GE on positive emotional states, depressive symptoms, fatigue perception during exercise, cardiac indicators, PA engagement, weight, body mass index, and body fat percentage, involving data from two trials. The results demonstrated a significant effect exclusively on post-activity perceived exertion: participants engaging in GE reported slightly less exertion compared to indoor exercise. However, PA characteristics varied among the three trials: running, a combination of strength and aerobic exercises, or cycling. Additionally, one trial included a clinical sample. A systematic review examined the long-term influence of PA in natural settings, including blue spaces, on quality of life (QL) (61). A meta-analysis of four randomised controlled trials did not find a significantly larger influence of green and blue exercise on general QL as measured by mental and physical health questionnaires. However, analysis based on six out of eight studies showed significant benefits of green and blue exercise activities on the psychological and physical health components of QL (61). Wicks et al. (62) found that only two out of twenty-four primary studies assessed the effects of repeated GE sessions, and no conclusive results about long-term effects were reported. In their review focusing on the long-term effects of PA interventions in green and blue space settings, Marini et al. (63) revealed that engagement in green and blue exercise yielded better responses in participants’ mood and well-being compared to exercising or resting in urban environments. However, this finding was based on two studies with differing characteristics. Vert et al. (64) investigated the long-term benefits of PA in blue spaces (a seafront route to a breakwater), while Song et al. (65) assessed the physiological and psychological short-term effects of GE by having participants walk in an urban park during the fall season. Overall, walking was the primary PA intervention in two out of the six studies included, while the remaining studies involved combined exercises such as body strength training and aerobic workouts.

### 3.3 Green space assessment

Fourteen studies, accounting for 20.1% of the total, evaluated the benefits of PA interventions in natural settings such as woodlands, grasslands, or forests, all featuring trails and footpaths. Then, interventions often took place in urban parks (15%) or university campuses (12%), with both environments varying in the extent of greenness. Eight studies (12%) picked environments with water elements, such as lakes and rivers. Outdoor exercise stations were utilised as GE in 6% of the studies. Another 12% of the studies involved indirect exposure to natural environments, using virtual reality and views of nature scenes. Other GE conditions included beaches, harbours, seas, hiking areas, urban public parks, and tree-lined urban roads.

#### 3.3.1 Objective environmental measures

Among the objective environmental measures, air temperature was the most reported (33.3%). Humidity was the second most frequently recorded (18.2%). Illumination levels (lux) were documented in 9.1% of studies, and noise levels were reported in 6.1% of studies. Wind speed, greenness rate, rainfall, sunlight exposure, and heat were less commonly measured.

#### 3.3.2 Subjective environmental measures

Regarding the subjective assessment, 9% of the studies utilised the Perceived Restorativeness Scale (PRS) (66) to evaluate the natural environment's restorative potential. Nisbet et al. (67) adapted the Positive and Negative Affect Scale (PANAS) by adding elements related to fascination, curiosity, and interest to assess the environment's restorative capacity. The semantic differential (SD) method was employed in 7.5% of studies to gauge participants’ environmental perceptions. One study examined the sense of presence in virtual green environments, while another study assessed perceived naturalness.

## 4 Discussion

This review aimed to synthesise current studies on the interplay between nature exposure and physical activity, focusing on methodological approaches and assessment metrics for the natural environment. Reviews on green exercise (GE) yielded mixed results, reflecting variability in short-term and long-term effects, likely influenced by diverse comparison methods, heterogeneous physical activity characteristics, and shortcomings in environmental assessment. Admittedly, this review has the limitation of focusing on a small number of reviews. The selection criteria led to a limited pool of studies, which may pose an issue of over-reliance on specific findings. However, the insights gained from this review provide essential guidance on key methodological issues in studying the benefits of green exercise and nature exposure.

The predominance of cross-sectional studies calls for longitudinal and robust experimental designs to unravel the complexities of the interrelation between exposure to nature and PA (56). Among the long-term effects of GE, a notable impact was observed only on perceived exertion levels (60) and sub-components of quality of life (61). However, inconsistent comparison conditions in these studies — such as resistance training with outdoor equipment or combined aerobic exercises as GE condition (60, 61, 63) — may hinder the generalizability of the long-term results.

Results also highlight overlooked aspects such as biodiversity in green settings (58) and the limited variety of green spaces utilised for PA (59). Reviewed studies often lacked objective nature measures and focused on subjective assessments by primary authors (58) or mixed green spaces and blue spaces (61, 63). Yen et al.'s (61) meta-analyses failed to demonstrate significantly greater benefits of GE on overall quality of life, potentially due to overlooked factors like aesthetic preferences and environmental variables. While Lahart et al. (60) found inconclusive evidence regarding the superiority of GE over non-green alternatives, short-term analyses showed the potential benefits on affective valence and enjoyment compared to indoor or non-green activities. Thus, methodological issues like poor descriptions of natural environments and limited subjective measures of environmental perceptions compromise the quality of evidence supporting GE.

Only a small portion (9%) of primary studies assessed the perceived restorative potential of natural settings, indicating a gap in understanding participants’ environmental preferences and perceptions. Nonetheless, perceptions of the physical environment are crucial for understanding the relationship between environment and PA (68), influencing engagement in GE (69, 70). For example, outdoor thermal comfort (OTC) can significantly affect how people perceive and use outdoor spaces, influencing participation in outdoor activities and overall urban livability, particularly in the context of climate change (71).

### 4.1 Future directions

Researching the efficacy of GE presents unique challenges, as it necessitates concurrent assessments of PA and nature exposure. An optimal approach involves monitoring PA levels through self-reports and contextually using device-based measures (48). Self-report tools, while valuable for exploring social and environmental aspects such as the perception of the activity's setting (72), should be complemented with objective methods to ascertain attitudes towards PA (52). Implementing standardised PA measurements is essential to combat the pandemic of physical inactivity (9) and pursue the WHO goal of reducing inactivity levels (3). As such, Ecological Momentary Assessment (EMA) allows for real-time collection and transmission of self-reported data on emotions, behaviours, and environmental perceptions, enabling the examination of environmental factors that contribute to PA maintenance (73).

Furthermore, future studies should explore various environmental features: the objective quality of physical settings, perceptions of restoration potential, perceived biodiversity, ecological quality, outdoor thermal comfort and aesthetic preference. In addition, future research should include meta-analyses to assess whether specific natural environments provide distinct health benefits. While the current literature often compares green spaces with urban or indoor environments, evaluating how different types of natural settings, such as urban parks vs. wilderness areas, affect health outcomes for participants engaging in physical activity would be valuable.

In addition to the health benefits of green exercise, future research should explore its potential to foster pro-environmental behaviours. Evidence suggests that regular contact with nature strengthens environmental awareness and encourages behaviours aimed at protecting the environment (74). Understanding how green exercise can promote individual well-being and pro-environmental attitudes could offer valuable insights for advancing public health and sustainability initiatives.

However, the ongoing impacts of climate change, including increased urban heat stress, may alter the efficacy of green and blue spaces in promoting health, with different types of green spaces potentially providing distinct benefits for mental health and climate resilience (2). In addition, the perception and use of green spaces can vary depending on urban context and climate. In warmer climates, high temperatures may discourage outdoor exercise, whereas in more temperate areas, green exercise tends to be more common (75). Thus, it is crucial that future research investigates how different types of natural environments may offer varied health benefits, especially in the context of changing climatic conditions.

### 4.2 Conclusion

The journey to quantify the health benefits of nature exposure is ongoing, and the field has yet to reach a consensus on the most predictive nature exposure measures (76). Future studies should aim for precision in evaluating these metrics’ accuracy and identifying specific natural elements for measurement (77). Current research often portrays natural spaces as static entities, primarily focusing on their visual aspects (45). This perspective may overlook the dynamic human-environment interaction over time *and* the nuanced impact of biodiversity and restoration potential on well-being (78).

The prevalent “green vs. urban” framework in studies of restorative environments may be too simplistic, potentially obscuring which specific environmental features confer health benefits (31). The ecological diversity within green spaces is a promising research area, as it is closely tied to the restorative benefits of nature exposure (79). Research has shown that higher biodiversity levels within urban and peri-urban green spaces contribute to increased perceptions of restorativeness and well-being, emphasising the importance of ecological diversity in promoting mental and physical health benefits (80). Furthermore, integrating regenerative urban design and biophilic principles into green spaces enhances their ability to foster healthful living while addressing climate resilience and sustainability (81). Thus, research that disentangles how different natural settings interact with PA to foster healthful living is a clear need for research.

This review highlights the multifaceted nature of GE and its effects on mental and physical health. While we understand some short-term benefits, the long-term impacts and the most effective environmental settings for promoting these benefits remain unclear. The contributions of subjective and objective measures of environmental attributes to GE outcomes require further investigation. Future research should integrate diverse methodologies, including EMA and advances in wearable technology, to capture the interplay of behaviour, environment, and time (72, 82). Moving forward, it is crucial to expand our metrics beyond “green” to encompass the broader spectrum of ecological features that enhance the restorative potential of natural environments. Only through a comprehensive understanding can we fully leverage the power of GE to combat physical inactivity and improve global health outcomes, as outlined by the WHO (3).

## References

[1] World Health Organization. Urban Green Spaces and Health: A Review of Evidence. Copenhagen: WHO Regional Office for Europe (2016).

[2] World Health Organization. Green and Blue Spaces and Mental Health: New Evidence and Perspectives for Action. Copenhagen: World Health Organization. Regional Office for Europe (2021). Available online at: https://apps.who.int/iris/handle/10665/342931

[3] Global Action Plan on Physical Activity 2018–2030: More Active People for a Healthier World. Geneva: World Health Organization (2018). Available online at: https://www.who.int/publications/i/item/9789241514187

[4] Warburton DER Nicol CW Bredin SSD. Health benefits of physical activity: the evidence. CMAJ. (2006) 174(6):801–9. 10.1503/cmaj.051351 16534088 PMC1402378

[5] World Health Organization. Considerations in Adjusting Public Health and Social Measures in the Context of COVID-19. Interim guidance. Geneva: WHO (2020). Available online at: https://www.who.int/publications/i/item/who-2019-ncov-adjusting-ph-measures-2023.1

[6] Bull FC Al-Ansari SS Biddle S Borodulin K Buman MP Cardon G World health organization 2020 guidelines on physical activity and sedentary behaviour. Br J Sports Med. (2020) 54(24):1451–62. 10.1136/bjsports-2020-102955 33239350 PMC7719906

[7] Teychenne M White RL Richards J Schuch FB Rosenbaum S Bennie JA. Do we need physical activity guidelines for mental health: what does the evidence tell US? Ment Health Phys Act. (2020) 18:100315. 1–5. 10.1016/j.mhpa.2019.100315

[8] Babyak M Blumenthal JA Herman S Khatri P Doraiswamy M Moore K Exercise treatment for major depression: maintenance of therapeutic benefit at 10 months. Psychosom Med. (2000) 62(5):633–8. 10.1097/00006842-200009000-00006 11020092

[9] Pratt M Ramirez Varela A Salvo D Kohl HW DIng D. Attacking the pandemic of physical inactivity: what is holding US back? Br J Sports Med. (2020) 54(13):760–2. 10.1136/bjsports-2019-101392 31704698

[10] Shanahan DF Bush R Gaston KJ Lin BB Dean J Barber E Health benefits from nature experiences depend on dose. Sci Rep. (2016) 6(28551):1–10. 10.1038/srep28551 27334040 PMC4917833

[11] van den Berg M Wendel-Vos W van Poppel M Kemper H van Mechelen W Maas J. Health benefits of green spaces in the living environment: a systematic review of epidemiological studies. Urban For Urban Green. (2015) 14(4):806–16. 10.1016/j.ufug.2015.07.008

[12] McMahan EA Estes D. The effect of contact with natural environments on positive and negative affect: a meta-analysis. J Posit Psychol. (2015) 10(6):507–19. 10.1080/17439760.2014.994224

[13] Maas J Verheij RA Groenewegen PP De Vries S Spreeuwenberg P. Green space, urbanity, and health: how strong is the relation? J Epidemiol Community Health. (2006) 60(7):587–92. 10.1136/jech.2005.043125 16790830 PMC2566234

[14] Kolokotsa D Lilli A Lilli MA Nikolaidis NP. On the impact of nature-based solutions on citizens’ health & well being. Energy Build. (2020) 229:110527. 1–31. 10.1016/j.enbuild.2020.110527

[15] Loureiro A Veloso S. Green exercise, health and well-being. In: Fleury-Bahi G Pol E Navarro O, editors. Handbook of Environmental Psychology and Quality of Life Research. Cham: Springer International Publishing AG (2017). p. 149–69. 10.1007/978-3-319-31416-7_8

[16] Pretty J Peacock J Sellens M Griffin M. The mental and physical health outcomes of green exercise. Int J Environ Health Res. (2005) 15(5):319–37. 10.1080/09603120500155963 16416750

[17] Rogerson M Barton J Pretty J Gladwell V. The green exercise concept. In: Donnelly AA MacIntyre TE, editors. Physical Activity in Natural Settings. London: Routledge. (2019). p. 75–94. 10.4324/9781315180144-4

[18] Markevych I Schoierer J Hartig T Chudnovsky A Hystad P Dzhambov AM Exploring pathways linking greenspace to health: theoretical and methodological guidance. Environ Res. (2017) 158:301–17. 10.1016/j.envres.2017.06.028 28672128

[19] Guerreiro CBB Foltescu V de Leeuw F. Air quality status and trends in Europe. Atmos Environ. (2014) 98:376–84. 10.1016/j.atmosenv.2014.09.017

[20] Hartig T Mitchell R De Vries S Frumkin H. Nature and health. Annu Rev Public Health. (2014) 35:207–28. 10.1146/annurev-publhealth-032013-182443 24387090

[21] Nowak DJ Hirabayashi S Bodine A Greenfield E. Tree and forest effects on air quality and human health in the United States. Environ Pollut. (2014) 193:119–29. 10.1016/j.envpol.2014.05.028 25016465

[22] Hartig T. Restorative environments. In: Spielberger C, editor. Encyclopedia of Applied Psychology. San Diego: Academic Press (2004) 3. p. 273–9.

[23] Von Lindern E Lymeus F Hartig T. The restorative environment: a complementary concept for salutogenesis studies. In: Mittelmark MB, editor. The Handbook of Salutogenesis. 1st ed Cham: Springer (2022). p. 371–95. 10.1007/978-3-030-79515-3_35 28590625

[24] Ulrich RS Simons RF Losito BD Fiorito E Miles MA Zelson M. Stress recovery during exposure to natural and urban environments. J Environ Psychol. (1991) 11(3):201–30. 10.1016/S0272-4944(05)80184-7

[25] Nukarinen T Rantala J Korpela K Browning MHEM Istance HO Surakka V Measures and modalities in restorative virtual natural environments: an integrative narrative review. Comput Human Behav. (2022) 126:107008. 1–14. 10.1016/j.chb.2021.107008

[26] Joye Y van den Berg AE. Restorative environments. In: Steg L de Groot JIM, editors. Environmental Psychology: An introduction. Hoboken, NJ: BPS Blackwell (2018). p. 57–66. 10.1002/9781119241072

[27] Kaplan S. The restorative benefits of nature: toward an integrative framework. J Environ Psychol. (1995) 15(3):169–82. 10.1016/0272-4944(95)90001-2

[28] Kellert SR Wilson EO. The Biophilia Hypothesis. Washington DC: Island Press (1993).

[29] Kaplan S Berman MG. Directed attention as a common resource for executive functioning and self-regulation. Perspect Psychol Sci. (2010) 5(1):43–57. 10.1177/1745691609356784 26162062

[30] Hartig T Evans GW Jamner LD Davis DS Gärling T. Tracking restoration in natural and urban field settings. J Environ Psychol. (2003) 23(2):109–23. 10.1016/S0272-4944(02)00109-3

[31] Berg Vd Jorgensen AE & Wilson A R E. Evaluating restoration in urban green spaces: does setting type make a difference? Landsc Urban Plann. (2014) 127:173–81. 10.1016/j.landurbplan.2014.04.012

[32] Joye Y Dewitte S. Nature’s broken path to restoration. A critical look at attention restoration theory. J Environ Psychol. (2018) 59:1–8. 10.1016/j.jenvp.2018.08.006

[33] Joye Y van den Berg A. Is love for green in our genes? A critical analysis of evolutionary assumptions in restorative environments research. Urban For Urban Green. (2011) 10(4):261–8. 10.1016/j.ufug.2011.07.004

[34] Joye Y Steg L Ünal AB Pals R. When complex is easy on the mind: internal repetition of visual information in complex objects is a source of perceptual fluency. J Exp Psychol Hum Percept Perform. (2016) 42(1):103–14. 10.1037/xhp0000105 26322692

[35] Reber R Schwarz N Winkielman P. Processing fluency and aesthetic pleasure: is beauty in the perceiver’s processing experience? Pers Soc Psychol Rev. (2004) 8(4):364–82. 10.1207/s15327957pspr0804_3 15582859

[36] Korpela KM Ylén M Tyrväinen L Silvennoinen H. Determinants of restorative experiences in everyday favorite places. Health Place. (2008) 14(4):636–52. 10.1016/j.healthplace.2007.10.008 18037332

[37] Hartig T. Restoration in nature: beyond the conventional narrative. In: Schutte AR Torquati JC Steven JR, editors. Nature and Psychology: Biological, Cognitive, Developmental, and Social Pathways to Well-being. Cham: Springer (2021). p. 89–151. 10.1007/978-3-030-69020-5

[38] James P Banay RF Hart JE Laden F. A review of the health benefits of greenness. Curr Epidemiol Rep. (2015) 2(2):131–42. 10.1007/s40471-015-0043-7 26185745 PMC4500194

[39] Collado S Staats H Corraliza JA Hartig T. Restorative environments and health. In: Fleury-Bahi G Pol E Navarro O, editors. Handbook of Environmental Psychology and Quality of Life Research. Cham: Springer International Publishing AG (2017). p. 127–48. 10.1007/978-3-319-31416-7_7

[40] Broekhuizen K de Vries SI Pierik FH. Healthy Aging in a Green Living Environment: A Systematic Review of the Literature. Leiden: TNO (2013). Available online at: https://publications.tno.nl/publication/101046/5zuNhA/broekhuizen-2013-healthy.pdf

[41] Lachowycz K Jones AP. Greenspace and obesity: a systematic review of the evidence. Obes Rev. (2011) 12(5):e183–9. 10.1111/j.1467-789X.2010.00827.x 21348919

[42] Shanahan DF Franco L Lin BB Gaston KJ Fuller RA. The benefits of natural environments for physical activity. Sports Med. (2016) 46(7):989–95. 10.1007/s40279-016-0502-4 26886475

[43] Gibson JJ. The Ecological Approach to Visual Perception: Classic Edition. 1st ed New York: Pscyhology Press (2014). 10.4324/9781315740218

[44] Araújo D Brymer E Brito H Withagen R Davids K. The empowering variability of affordances of nature: why do exercisers feel better after performing the same exercise in natural environments than in indoor environments? Psychol Sport Exerc. (2019) 42(2019):138–45. 10.1016/j.psychsport.2018.12.020

[45] Bamberg J Hitchings R Latham A. Enriching green exercise research. Landsc Urban Plann. (2018) 178:270–5. 10.1016/j.landurbplan.2018.06.005

[46] Yeh HP Stone JA Churchill SM Wheat JS Brymer E Davids K. Physical, psychological and emotional benefits of green physical activity: an ecological dynamics perspective. Sports Med. (2016) 46(7):947–53. 10.1007/s40279-015-0374-z 26330207

[47] Barton J Wood C Pretty J Rogerson M. Green exercise for health: a dose of nature. In: Barton J Bragg R Wood C Pretty J, editors. Green Exercise: Linking Nature, Health and Well-being. 1st ed. New York, NY: Routledge (2016). p. 26–36. 10.4324/9781315750941-9

[48] Ding D Ramirez Varela A Bauman AE Ekelund U Lee IM Heath G Towards better evidence-informed global action: lessons learnt from the lancet series and recent developments in physical activity and public health. Br J Sports Med. (2020) 54(8):462–8. 10.1136/bjsports-2019-101001 31562122 PMC7146932

[49] Craig CL Marshall AL Sjöström M Bauman AE Booth ML Ainsworth BE International physical activity questionnaire: 12-country reliability and validity. Med Sci Sports Exercise. (2003) 35(8):1381–95. 10.1249/01.MSS.0000078924.61453.FB 12900694

[50] Cerin E Cain KL Oyeyemi AL Owen N Conway TL Cochrane T Correlates of agreement between accelerometry and self-reported physical activity. Med Sci Sports Exercise. (2016) 48(6):1075–84. 10.1249/MSS.0000000000000870 PMC4868646 26784274

[51] Nelson MC Taylor K Vella CA. Comparison of self-reported and objectively measured sedentary behavior and physical activity in undergraduate students. Meas Phys Educ Exerc Sci. (2019) 23(3):237–48. 10.1080/1091367X.2019.1610765 31903020 PMC6941744

[52] Steene-Johannessen J Anderssen SA Van Der Ploeg HP Hendriksen IJM Donnelly AE Brage S Are self-report measures able to define individuals as physically active or inactive? Med Sci Sports Exercise. (2016) 48(2):235–44. 10.1249/MSS.0000000000000760 PMC6235100 26322556

[53] Holland I Deville NV Browning MHEM Buehler RM Hart JE Aaron Hipp J Measuring nature contact: a narrative review. Int J Environ Res Public Health. (2021) 18(40092):4092–15. 10.3390/ijerph18084092 33924490 PMC8069863

[54] Pettorelli N Vik JO Mysterud A Gaillard JM Tucker CJ Stenseth NC. Using the satellite-derived NDVI to assess ecological responses to environmental change. Trends Ecol Evol. (2005) 20(9):503–10. 10.1016/j.tree.2005.05.011 16701427

[55] Lu Y. Using google street view to investigate the association between street greenery and physical activity. Landsc Urban Plann. (2019) 191:103435. 1–9. 10.1016/j.landurbplan.2018.08.029

[56] Remme RP Frumkin H Guerry AD King AC Mandle L Sarabu C An ecosystem service perspective on urban nature, physical activity, and health. Proc Natl Acad Sci U S A. (2021) 118(22):e2018472118. 1–10. 10.1073/pnas.2018472118 33990458 PMC8179134

[57] Pollock M Fernandes RM Becker LA Pieper D Hartling L. Chapter V: overviews of reviews. In: Higgins JPT Thomas J Chandler J Cumpston M Li T Page MJ Welch VA, editors. Cochrane Handbook for Systematic Reviews of Interventions (Version 6.5). Chichester: John Wiley and Sons (2024). Available online at: www.training.cochrane.org/handbook

[58] Bowler DE Buyung-Ali LM Knight TM Pullin AS. A systematic review of evidence for the added benefits to health of exposure to natural environments. BMC Public Health. (2010) 10:1–10. 10.1186/1471-2458-10-456 20684754 PMC2924288

[59] Thompson Coon J Boddy K Stein K Whear R Barton J Depledge MH. Does participating in physical activity in outdoor natural environments have a greater effect on physical and mental wellbeing than physical activity indoors? A systematic review. Environ Sci Technol. (2011) 45(5):1761–72. 10.1021/es102947t 21291246

[60] Lahart I Darcy P Gidlow C Calogiuri G. The effects of green exercise on physical and mental wellbeing: a systematic review. Int J Environ Res Public Health. (2019) 16(8):1352. 1–26. 10.3390/ijerph16081352 30991724 PMC6518264

[61] Yen HY Chiu HL Huang HY. Green and blue physical activity for quality of life: a systematic review and meta-analysis of randomized control trials. Landsc Urban Plann. (2021) 212:104093. 1–9. 10.1016/j.landurbplan.2021.104093

[62] Wicks C Barton J Orbell S Andrews L. Psychological benefits of outdoor physical activity in natural versus urban environments: a systematic review and meta-analysis of experimental studies. Appl Psychol Health Well Being. (2022) 14(3):1037–61. 10.1111/aphw.12353 35259287 PMC9544808

[63] Marini S Mauro M Grigoletto A Toselli S Maietta Latessa P. The effect of physical activity interventions carried out in outdoor natural blue and green spaces on health outcomes: a systematic review. Int J Environ Res Public Health. (2022) 19(19):12482. 1–15. 10.3390/ijerph191912482 36231779 PMC9566520

[64] Vert C Gascon M Ranzani O Márquez S Triguero-Mas M Carrasco-Turigas G Physical and mental health effects of repeated short walks in a blue space environment: a randomised crossover study. Environ Res. (2020) 188:109812. 1–15. 10.1016/j.envres.2020.109812 32590148

[65] Song C Ikei H Igarashi M Takagaki M Miyazaki Y. Physiological and psychological effects of a walk in urban parks in fall. Int J Environ Res Public Health. (2015) 12(11):14216–28. 10.3390/ijerph121114216 26569271 PMC4661642

[66] Pasini M Berto R Brondino M Hall R Ortner C. How to measure the restorative quality of environments: the PRS-11. Proc Soc Behav Sci. (2014) 159:293–7. 10.1016/j.sbspro.2014.12.375

[67] Nisbet EK Zelenski JM. Underestimating nearby nature: affective forecasting errors obscure the happy path to sustainability. Psychol Sci. (2011) 22(9):1101–6. 10.1177/0956797611418527 21828351

[68] Brownson RC Hoehner CM Day K Forsyth A Sallis JF. Measuring the built environment for physical activity. Am J Prev Med. (2009) 36(4 Supplment):S99–S123.e12. 10.1016/j.amepre.2009.01.005 19285216 PMC2844244

[69] Menardo E De Dominicis S Pasini M. Exploring perceived and objective measures of the neighborhood environment and associations with physical activity among adults: a review and a meta-analytic structural equation model. Int J Environ Res Public Health. (2022) 19(5):2575. 1–15. 10.3390/ijerph19052575 35270267 PMC8909183

[70] Petrunoff NA Edney S Yi NX Dickens BL Joel KR Xin WN Associations of park features with park use and park-based physical activity in an urban environment in Asia: a cross-sectional study. Health Place. (2022) 75:102790. 1–11. 10.1016/j.healthplace.2022.102790 35316722

[71] Chen L Ng E. Outdoor thermal comfort and outdoor activities: a review of research in the past decade. Cities. (2012) 29(2):118–25. 10.1016/j.cities.2011.08.006

[72] Sattler MC Ainsworth BE Andersen LB Foster C Hagströmer M Jaunig J Physical activity self-reports: past or future? Br J Sports Med. (2021) 55(16):889–90. 10.1136/bjsports-2020-103595 33536193 PMC8477753

[73] Rhodes RE McEwan D Rebar AL. Theories of physical activity behaviour change: a history and synthesis of approaches. Psychol Sport Exerc. (2019) 42:100–9. 10.1016/j.psychsport.2018.11.010

[74] Mackay CML Schmitt MT. Do people who feel connected to nature do more to protect it? A meta-analysis. J Environ Psychol. (2019) 65:101323. 1–9. 10.1016/j.jenvp.2019.101323

[75] Delgado-Serrano MM Melichová K Mac Fadden I Cruz-Piedrahita C. Perception of green spaces’ role in enhancing mental health and mental well-being in small and medium-sized cities. Land Use Policy. (2024) 139: 107087. 1–11. 10.1016/j.landusepol.2024.107087

[76] MacIntyre TE Calogiuri G Donnelly AA Warrington G Beckmann J Lahart I Societal challenges, methodological issues and transdisciplinary approaches. In: Donnelly AA MacIntyre TE, editors. Physical Activity in Natural Settings. 1st ed. London: Routledge (2019). p. 15–35. 10.4324/9781315180144-2

[77] Frumkin H Bratman GN Breslow SJ Cochran B Kahn PH Lawler JJ Nature contact and human health: a research agenda. Environ Health Perspect. (2017) 125(7):075001.1–075001.18. 10.1289/EHP1663 28796634 PMC5744722

[78] Fuller RA Irvine KN Devine-Wright P Warren PH Gaston KJ. Psychological benefits of greenspace increase with biodiversity. Biol Lett. (2007) 3(4):390–4. 10.1098/rsbl.2007.0149 17504734 PMC2390667

[79] Wood E Harsant A Dallimer M de Chavez AC McEachan RRC Hassall C. Not all green space is created equal: biodiversity predicts psychological restorative benefits from urban green space. Front Psychol. (2018) 9:2320. 1–13. 10.3389/fpsyg.2018.02320 30538653 PMC6277587

[80] Carrus G Scopelliti M Lafortezza R Colangelo G Ferrini F Salbitano F Go greener, feel better? The positive effects of biodiversity on the well-being of individuals visiting urban and peri-urban green areas. Landsc Urban Plann. (2015) 134:221–8. 10.1016/j.landurbplan.2014.10.022

[81] Lencastre MPA Vidal DG Lopes HS Curado MJ. Biophilia in pieces: critical approach of a general concept. Environ Soc Psychol. (2023) 8(3):1. 10.54517/esp.v8i3.1869

[82] Dunton GF. Ecological momentary assessment in physical activity research. Exerc Sport Sci Rev. (2017) 45(1):48–54. 10.1249/JES.0000000000000092 27741022 PMC5161656

